# How manure amendment counters no-tillage yield reduction in winter wheat: enhanced nitrogen components and soil enzyme activity

**DOI:** 10.3389/fpls.2026.1834070

**Published:** 2026-06-11

**Authors:** Wenbo Mi, Shuying Wang, Jianjun Zhang, Gang Zhao, Yi Dang, Lei Wang, Gang Zhou, Xujiao Zhou, Jingyu Hu, Shangzhong Li, Daozhi Gong, Tinglu Fan

**Affiliations:** 1 State Key Laboratory of Efficient Utilization of Agricultural Water Resources, China Agricultural University, Beijing, China; 2 Institute of Dryland, Gansu Academy of Agricultural Sciences, Lanzhou, China; 3 Key Laboratory of Low Carbon Green Agriculture in Northwestern China, Ministry of Agriculture and Rural Affairs, Lanzhou, China; 4 Key Laboratory of Higher Water Utilization on Dryland of Gansu Province, Lanzhou, China

**Keywords:** carbon and nitrogen fractions, no-tillage, organic fertilizer, soil enzyme, yield

## Abstract

**Introduction:**

No-tillage (NT) effectively reduces soil erosion, enhances water retention, and promotes carbon sequestration, yet it often leads to yield reduction. The application of manure can mitigate the yield decline caused by NT. Therefore, combining NT with manure represents a key strategy for achieving sustainable agriculture. This study aimed to evaluate the effects of organic fertilizer application on soil physicochemical properties in long-term NT farmland, and reveal the regulatory associations of manure on crop yield in such systems.

**Methods:**

A split-plot design was employed, with tillage practices (CT and NT) as the main plots and fertilization treatments as sub-plots. The fertilization treatments included: no fertilizer (CK), mineral nitrogen (N), mineral phosphorus (P), manure-only (M); mineral N and P (NP); and combined N, P, and manure (MNP).

**Results:**

Compared with CT, NT significantly increased soil carbon fractions (SOC, and MBC by 19.09%, and 14.95%, respectively) and nitrogen fractions (AN and NN by 35.48% and 67.82%, respectively), as well as BD (increased by 11.83%), but decreased soil enzyme activities (CBH and NAG by 9.40% and 9.09%, respectively). In contrast, the combined application of manure increased soil carbon/nitrogen fractions, enzyme activities while effectively reducing soil BD and pH. Specifically, compared with the NP, the MNP treatment increased SOC, TN, AP, AN, CBH, and NAG by 31.25%, 23.67%, 65.35%, 28.48%, 37.33%, and 30.79%, respectively. Under the MNP, yield increased by 9.84% (CT) and 13.40% (NT) compared to the NP treatment. Regression and correlation analyses showed that soil enzyme activities were positively correlated with soil nutrients.

**Discussion:**

The interaction between NT and manure application regulated soil physical structure, thereby enhancing the activities of enzymes related to carbon and nitrogen cycling. This led to a significant increase in carbon and nitrogen storage, ultimately improving crop yield. The continuous input of manure and the improved availability of nitrogen fractions were identified as the core factors contributing to the yield gain. This study confirms that no-tillage combined with manure is an effective strategy for coordinating soil carbon sequestration and stable crop yield improvement, providing a feasible management optimization approach for the promotion of no-tillage technology.

## 1 Introduction

No-tillage (NT), as the core of conservation agriculture, is gaining increasing global importance. By avoiding soil turnover and maintaining surface coverage, it minimizes soil and water erosion, enhances soil water retention, and promotes the sequestration of soil organic carbon. It holds irreplaceable long-term value in addressing global land degradation, water scarcity, and climate change mitigation. However, NT can lead to increased soil compaction, lower pre-sowing temperatures, and challenges in weed control, ultimately resulting in reduced crop yields (Dou et al., 2024). Appropriate field management practices (straw return, organic fertilizer application, precision fertilization, and diversified crop rotation) can offset the yield-reducing effects of no-till farming. however, these measures do not yield immediate results (Lv et al., 2024). Therefore, conducting long-term field trials to validate these findings is of great significance for the sustainable development of global agriculture (Jin et al., 2024; Zhang et al., 2025).

Soil is the critical foundation for crop survival. Soil health affects food production, environmental services, and adaptive capacity (Liu et al., 2025). However, inappropriate fertilization practices are threatening soil quality and the functionality of farmland systems, leading to a series of soil issues. For example, excessive fertilization can cause nutrient imbalance, surface compaction, and changes in microbial community structure, ultimately resulting in reduced crop yields (Hu et al., 2026; Sun et al., 2025). Soil carbon and nitrogen components play an indispensable role in assessing soil health. Soil carbon components (such as SOC, MBC, DOC, etc.) serve as both drivers of soil nutrient cycling and sensitive indicators of soil health. They provide the energy required for microbial activity and drive the mineralization and release of nutrients such as nitrogen and phosphorus. Their levels determine the short-term nutrient supply capacity of the soil (Ni et al., 2025). Additionally, particulate organic carbon acts as a binding agent in the formation of soil aggregates, directly influencing soil porosity and bulk density (Liu et al., 2025; Weidhuner et al., 2026). Thus, soil carbon components improve soil physical and biological properties as well as nutrient supply capacity, creating a foundational environment for high crop yields (Xuan et al., 2009). Soil nitrogen components are directly related to crop yield formation. Organic nitrogen (such as TN, MBN, etc.) is a vital nitrogen source for soil microorganisms, and its mineralization process is the fundamental pathway for soil nitrogen supply. Inorganic nitrogen (such as NH_4_^+^-N, NO_3_^--^N, etc.) directly controls crop growth rates and grain yield, serving as key nutrients for crop production (Malhi et al., 2006). Among them, NH_4_^+^-N plays a significant role in soil cation exchange and helps maintain soil pH buffering capacity. High yields rely on the synergistic relationship between carbon and nitrogen components (Bender et al., 2014; Song et al., 2025). Therefore, coordinated management strategies that “stabilize carbon, boost nitrogen, and use carbon to retain nitrogen”-such as combined organic and inorganic fertilization, and straw return-represent the optimal pathway for achieving high efficiency, productivity, and enhanced soil fertility.

The application of organic fertilizer not only provides the soil with comprehensive mineral nutrients and abundant organic matter but also expands the soil’s organic nitrogen reserves (Sun et al., 2025). On the one hand, organic fertilizer enhances the soil’s nutrient supply capacity and accelerates the conversion of nutrients from organic forms into available forms. On the other hand, it strengthens the soil’s ability to retain nutrients and sequester carbon, promoting the transformation of simple organic compounds into humus (Tian et al., 2022; Wang et al., 2024). This series of transformations primarily relies on soil extracellular enzymes to accomplish. Soil extracellular enzyme activity serves as an important indicator for assessing soil health and plays a key role in the cycling of soil carbon, nitrogen, and phosphorus. Soil microorganisms secrete these enzymes to decompose organic compounds (Borase et al., 2020; Elhawat et al., 2025; Yu et al., 2026). For instance, β-glucosidase (BG) and cellobiohydrolase (CBH), which are associated with soil carbon cycling, are the primary enzymes catalyzing cellulose degradation. N-acetylglucosaminidase (NAG) and leucine aminopeptidase (LAP) are involved in soil nitrogen metabolism. NAG hydrolyzes N-acetylglucosamine in chitobiose and peptidoglycan, a major component of bacterial cell walls. LAP, acting as a protease/peptidase, catalyzes the release of amino acids from proteins or other peptide substrates (Caldwell, 2005). Alkaline phosphatase (ALP) participates in soil phosphorus metabolism by hydrolyzing various phosphate esters to release phosphate ions, thereby influencing the cycling of soil carbon, nitrogen, and phosphorus (Elhawat et al., 2025; Yu et al., 2026). As an easily decomposable organic substrate, organic fertilizer can stimulate microbial activity and promote the secretion of extracellular enzymes, thereby enhancing their activity. At the same time, soil extracellular enzymes (such as CBH and NAG) play a key role in driving nitrogen transformation by degrading organic substrates, thereby regulating the availability of nitrogen components that directly affect crop yields. Numerous studies have shown that the application of organic fertilizers can stimulate enzyme activity and increase nitrogen availability, ultimately enhancing productivity (Yu et al., 2026; Zhang et al., 2026). However, in long-term NT systems, soil compaction and reduced enzyme aktivitas often lead to yield losses, and the relationship among enzyme aktivitas, nitrogen fractions, and yield remains poorly understood.

Therefore, we conducted a study on a farmland in Gansu Province, China, which had undergone continuous NT and fertilization for 20 years. The field experiment involved two tillage practices and six different fertilization treatments. By measuring indicators such as soil carbon fractions, nitrogen fractions, enzyme activities, and soil physical properties under different treatments, this study aimed to: (1) evaluate the effects of long-term NT and organic fertilizer application on soil physicochemical properties and enzyme activities; (2) clarify the intrinsic relationships between soil carbon and nitrogen fractions and enzyme activities under different tillage and fertilization treatments; and (3) reveal the regulatory mechanisms of organic fertilizer on crop yield in long-term NT farmland.

## 2 Materials and methods

### 2.1 Site description

**Figure 1:**
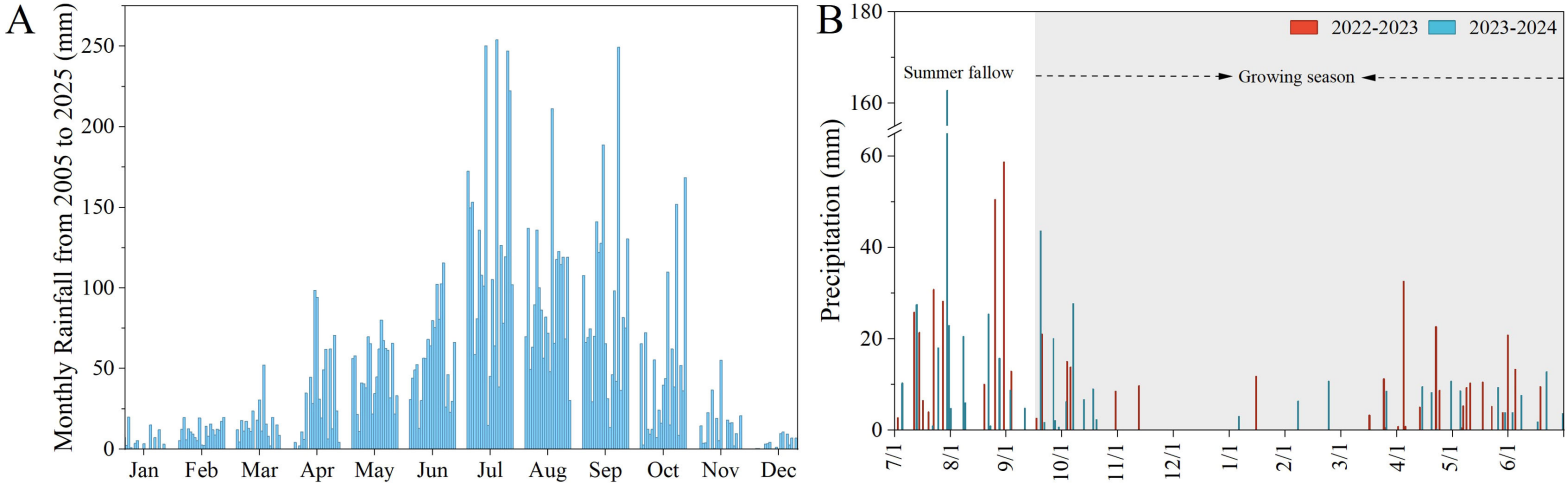
Precipitation in the experimental area from 2005 to 2025 **(A)** and precipitation during the winter wheat growing season from 2002 to 2004 **(B)**.

The long-term tillage and fertilization experiment was established in 2005 at the Northwest Dryland Nutrition and Fertilization Science Observation and Experiment Station of the Ministry of Agriculture and Rural Affairs, located in Wutong Village, Shangxiao Town, Zhenyuan County, Qingyang City, Gansu Province, on the Loess Plateau of eastern Gansu (35°29′42″ N, 107°29′36″ E; altitude 1279 m). The region has a warm temperate semi-arid continental monsoon climate and is a typical rain-fed agricultural area. Over the past 30 years, the mean annual precipitation has been 510 mm, with over 60% occurring from July to September, showing high interannual and seasonal variability. The annual potential evaporation is 1532 mm, the mean annual temperature is 9.3°C, and the frost-free period lasts 170 days. The initial soil chemical properties in the 0–40 cm layer were as follows: organic matter 14.46 g kg^-1^, total nitrogen 1.11 g kg^-1^, total phosphorus 0.77 g kg^-1^, total potassium 28.22 g kg^-1^, alkali-hydrolyzable nitrogen 78.7 mg kg^-1^, available phosphorus 13.9 mg kg^-1^, and available potassium 150 mg kg^-1^. The monthly rainfall from 2005 to 2025, as well as the rainfall and temperature variations during the wheat growing seasons from 2022 to 2024 are shown in **Figure 1**. Manuscript abbreviations in **Table 1**.

**Table 1:** Abbreviations list.

Abbreviations	Full name of indicator
NT	No-tillage
CT	Conventional tillage
SOC	Soil organic carbon
DOC	Dissolved Organic Carbon
MBC	Microbial biomass carbon
TN	Total nitrogen
NO_3_^--^N	Nitrate nitrogen
NH_4_^+^-N	Ammonium nitrogen
MBN	Microbial biomass nitrogen
TP	Total phosphorus
AP	Available phosphorus
AK	Available potassium
pH	Potential of hydrogen
BD	Bulk density
TSP	Total soil porosity
FWC	Field capacity
BG	β-Glucosidase
CBH	Cellobiose Hydrolase
NAG	N-Acetylglucosaminidase
LAP	Leucine Aminopeptidase
ALP	Alkaline Phosphatase
Y	Wheat yield
TSW	Thousand-seed weight

### 2.2 Experimental design

**Figure 2:**
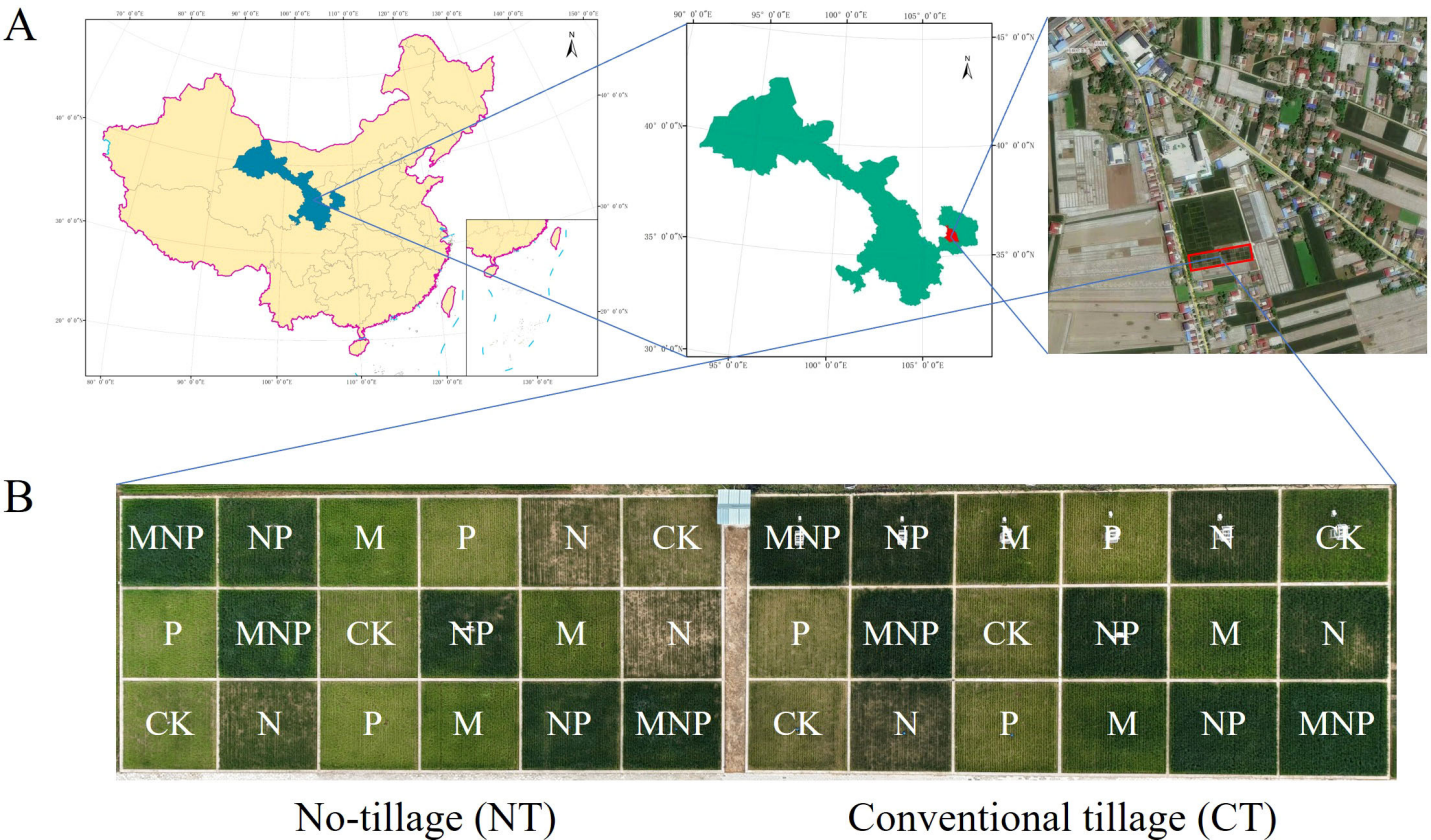
Specific location of the experimental area **(A)** and the field cropping system **(B)**.

The experiment employed a split-plot design. The main treatment was tillage method, consisting of conventional tillage (CT) and NT. CT involved one deep plowing after crop harvest and one rotary tillage before seeding. NT involved no soil disturbance from post-harvest to pre-sowing. Field weeds were controlled manually and chemically. The sub-treatment was fertilization, with the following six treatments: no-fertilizer control (CK); chemical nitrogen fertilizer (N 150 kg ha^-1^, N); phosphorus fertilizer (P_2_O_5_ 105 kg ha^-1^, P); manure fertilizer (decomposed pure cattle manure 22,500 kg ha^-1^, M); combined nitrogen and phosphorus fertilizer (N 150 kg ha^-1^ + P_2_O_5_ 105 kg ha^-1^, NP); and combined nitrogen, phosphorus fertilizer, and manure fertilizer (N 150 kg ha^-1^ + P_2_O_5_ 105 kg ha^-1^ + manure fertilizer 22,500 kg ha^-1^, MNP). The nutrient content of the manure was as follows: organic matter 15.2%, nitrogen 0.43%, phosphorus 0.45%, and potassium 0.28%. Each plot area was 72 m² (8 m × 9 m), with three replicates. The basal-to-topdressing ratio for nitrogen fertilizer was 5:5. For winter wheat, topdressing was manually broadcast during the greening stage following rainfall. For spring maize, topdressing was manually applied in holes at the jointing stage. Manure and phosphorus fertilizer were applied once as basal fertilizer before sowing. Under conventional tillage, fertilizers were incorporated during pre-sowing rotary tillage. Under NT, fertilizers were applied on the sowing day by being placed in furrows and harrowed in together with the seeds (approx. 5 cm deep). The crop rotation followed a sequence of one year of spring maize followed by three years of winter wheat. To minimize experimental error due to varietal differences, a uniform winter wheat cultivar, “Longjian 301”, was used with a seeding rate of 157.5 kg ha^-1^, sown manually in furrows with a row spacing of 20 cm. Winter wheat cultivars are characterized by strong stress resistance and high yield potential. For both CT and NT, winter wheat stubble height was maintained at approximately 3–5 cm, with roots allowed to decompose naturally in the field, while the remaining aboveground straw was completely removed. Under CT, wheat stubble was plowed into the soil. Under NT, wheat stubble was left on the soil surface. All other management practices followed conventional requirements. The manuscript utilizes experimental data from the wheat seasons of 2022–2023 and 2023-2024 (**Figure 2**).

### 2.3 Soil physical and chemical property analysis

At winter wheat harvest in 2023 and 2024. In each plot, soils were sampled at five spots with a soil auger, with visible stones removed and plant residues. After thorough mixing, a portion was stored at 4 °C for determining soil enzyme activity, microbial biomass carbon (MBC), microbial biomass nitrogen (MBN), nitrate nitrogen (NO_3_^--^N), and ammonium nitrogen (NH_4_^+^-N) content. The remaining soil sample was air-dried and sieved through a 2 mm mesh for soil nutrient content analysis. Soil total nitrogen (TN) and soil organic carbon (SOC) and were measured using an EA3000 elemental analyzer (Euro Vector, Italy). Soil-dissolved organic carbon (DOC) was determined using a total carbon analyzer. Soil total phosphorus (TP), available phosphorus (AP), and available potassium (AK) were determined following the methods described (Chen et al., 2023; Zhang et al., 2021). NH_4_^+^-N and NO_3_^--^N were extracted with potassium chloride (Wang et al., 2025). MBC and MBN were measured using the chloroform fumigation-extraction method (Zhang et al., 2021). Soil pH was measured in a 1:2.5 soil-to-water ratio using a digital pH meter. Soil bulk density (BD), Total soil porosity (TSP), and Field capacity (FWC) were determined using the cutting ring method (with a volume of 100 cm^3^) followed by oven-drying (Liu et al., 2025).

### 2.4 Soil enzyme activity analysis

The activities of BG and CBH associated with soil carbon metabolism, NAG and LAP related to soil nitrogen metabolism, and ALP involved in soil phosphorus metabolism were all determined using the microplate fluorescence method (Yu et al., 2026).

### 2.5 Wheat yield

At the maturity stage, wheat yield was measured by harvesting the entire plot after removing the outer 50 cm of rows. The wheat was weighed to calculate yield in kg·ha^-1^.

### 2.6 Statistical analysis

Two-way analysis of variance (ANOVAs) was performed to assess the effects of different treatments on soil physicochemical properties and enzyme activities. Differences among treatments were further evaluated using Fisher’s LSD *post-hoc* tests with multiple comparisons. Mantel tests were conducted in RStudio using the corrplot, tidyverse, and ggplot2 packages, while partial least squares path model (PLS-PM) was carried out with the plspm, pls, readx1, caret, and dplyr packages. All figures were generated using Origin 2021 and R 4.4.2 (https://cran.r-project.org).

## 3 Results

### 3.1 Soil carbon components

**Figure 3:**
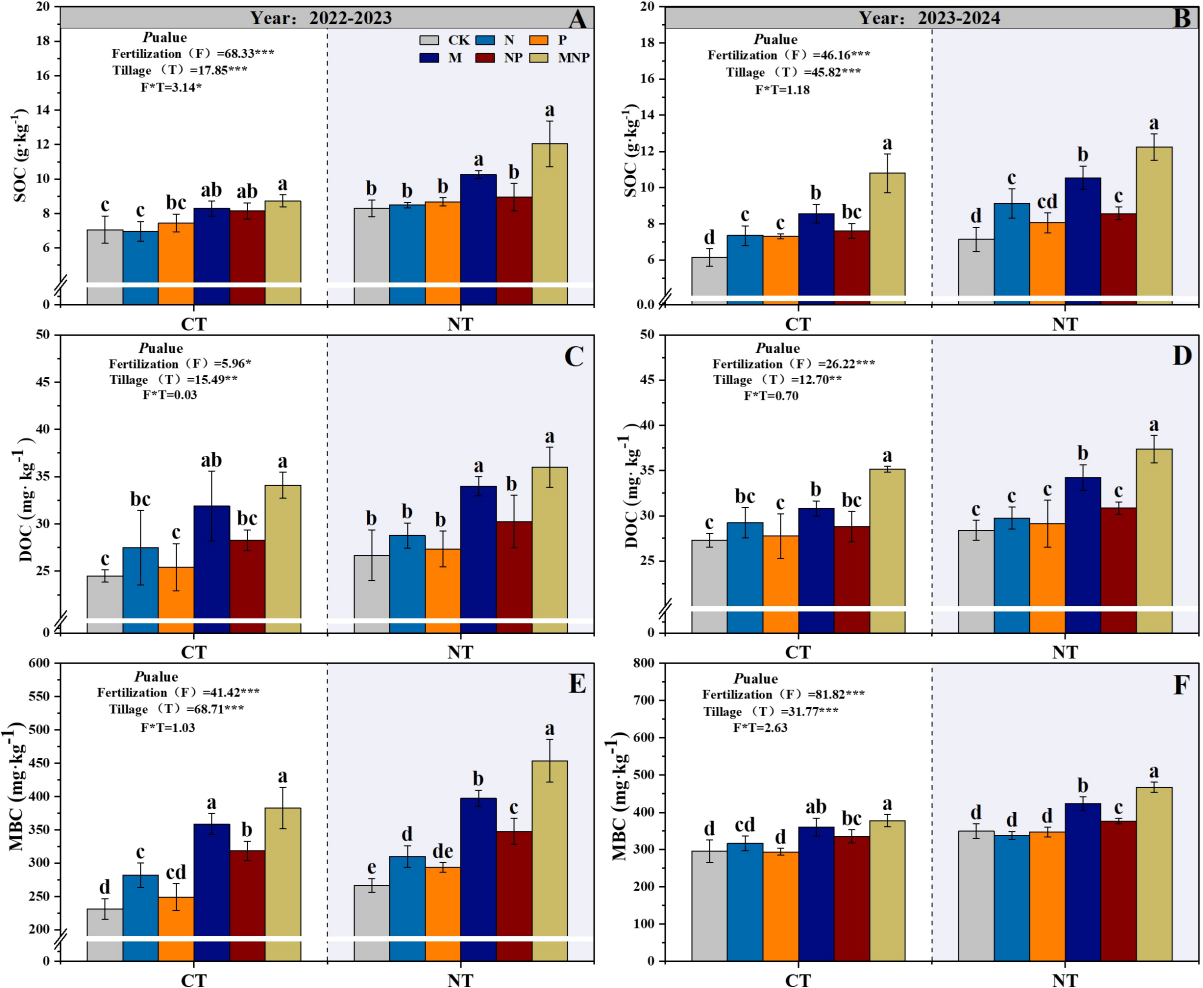
Response of soil carbon components under different treatments during the 2022–2023 and 2023–2024. Data are the means ± standard deviations (SD) (n = 3). Error bars depict standard deviations. Different letters indicate significant difference at *P* < 0.05 for treatments. ANOVA indicates ***, **, * indicates significance at 0.001, 0.01, 0.05 probability level, respectively. The same below. **(A, B)** SOC, soil organic carbon. **(C, D)** DOC, dissolved organic carbon. **(E, F)** MBC, microbial biomass carbon. Significant differences among treatments are indicated in different lowercase letters (P<0.05).

NT and fertilizer application (M and MNP) significantly increased soil carbon components (SOC, DOC, and MBC; *P* < 0.05). Compared to CT, the NT treatment increased SOC (**Figures 3A, B**), DOC (**Figures 3C, D**), and MBC (**Figures 3E, F**), by 19.09%, 14.95%, and 1.42%, respectively. Relative to the NP treatment, the MNP and M treatments increased SOC by 31.64% and 13.13%, DOC by 20.69% and 10.82%, and MBC by 64.58% and 33.23%, respectively. Furthermore, the order of SOC levels under different fertilization practices was MNP > M > NP > P > N > CK, while the order for DOC and MBC was MNP > M > NP > N > P > CK.

### 3.2 Soil nitrogen components

**Figure 4:**
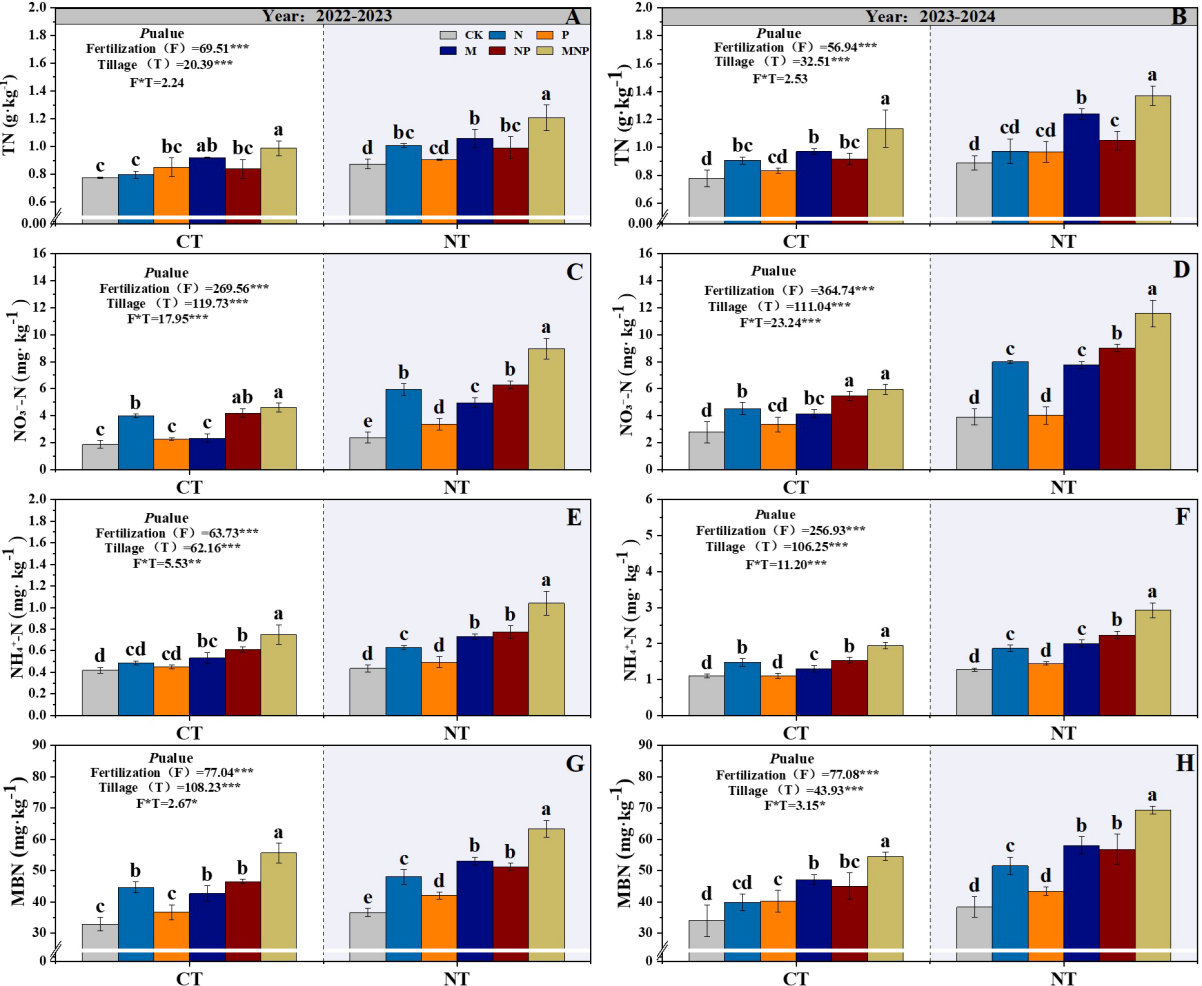
Response of soil nitrogen components under different treatments during the 2022–2023 and 2023–2024. **(A, B)** TN, total nitrogen. **(C, D)** NO_3_^−^-N, nitrate nitrogen. **(E, F)** NH_4_^+^-N, ammonium nitrogen. **(G, H)** MBN, microbial biomass nitrogen. Significant differences among treatments are indicated in different lowercase letters (P<0.05). *P<0.05, **P<0.01, ***P<0.001.

Both fertilization and tillage practices exerted highly significant effects on soil nitrogen components (TN, NO_3_^--^N, NH_4_^+^-N, and MBN; *P* < 0.001). Among them, the NT treatment increased TN (**Figures 4A, B**), NO_3_^—^N (**Figures 4C, D**), NH_4_^+^-N (**Figures 4E, F**), and MBN (**Figures 4G, H**) by 17.05%, 67.82%, 35.48%, and 17.77%, respectively, compared to CT. Manure application (M and MNP) effectively enhanced the accumulation of TN and MBN: under the MNP and M treatments, TN increased by 23.88% and 10.36%, and MN increased by 21.71% and 0.68%, respectively, relative to the NP treatment. The order of TN and MBN levels under different fertilization practices was MNP > M > NP > N > P > CK, while the order for NO_3_^--^N and NH_4_^+^-N was MNP > NP > N > M > P > CK. This discrepancy may be attributed to the fact that the order of total nitrogen is determined by the accumulation of organic nitrogen. The M and MNP treatments significantly expanded the organic nitrogen pool through long-term immobilization, whereas the fertilizer-only treatments resulted in less organic nitrogen accumulation. In contrast, the order of mineral nitrogen is governed by the net mineralization rate. Although the M treatment had high total nitrogen, its organic nitrogen mineralized slowly and may have undergone net immobilization, leading to relatively low mineral nitrogen levels. Compared to the NP treatment, the MNP treatment increased NN and AN by 24.45% and 28.99%, respectively.

### 3.3 Total phosphorus, available phosphorus, available potassium and pH

**Figure 5:**
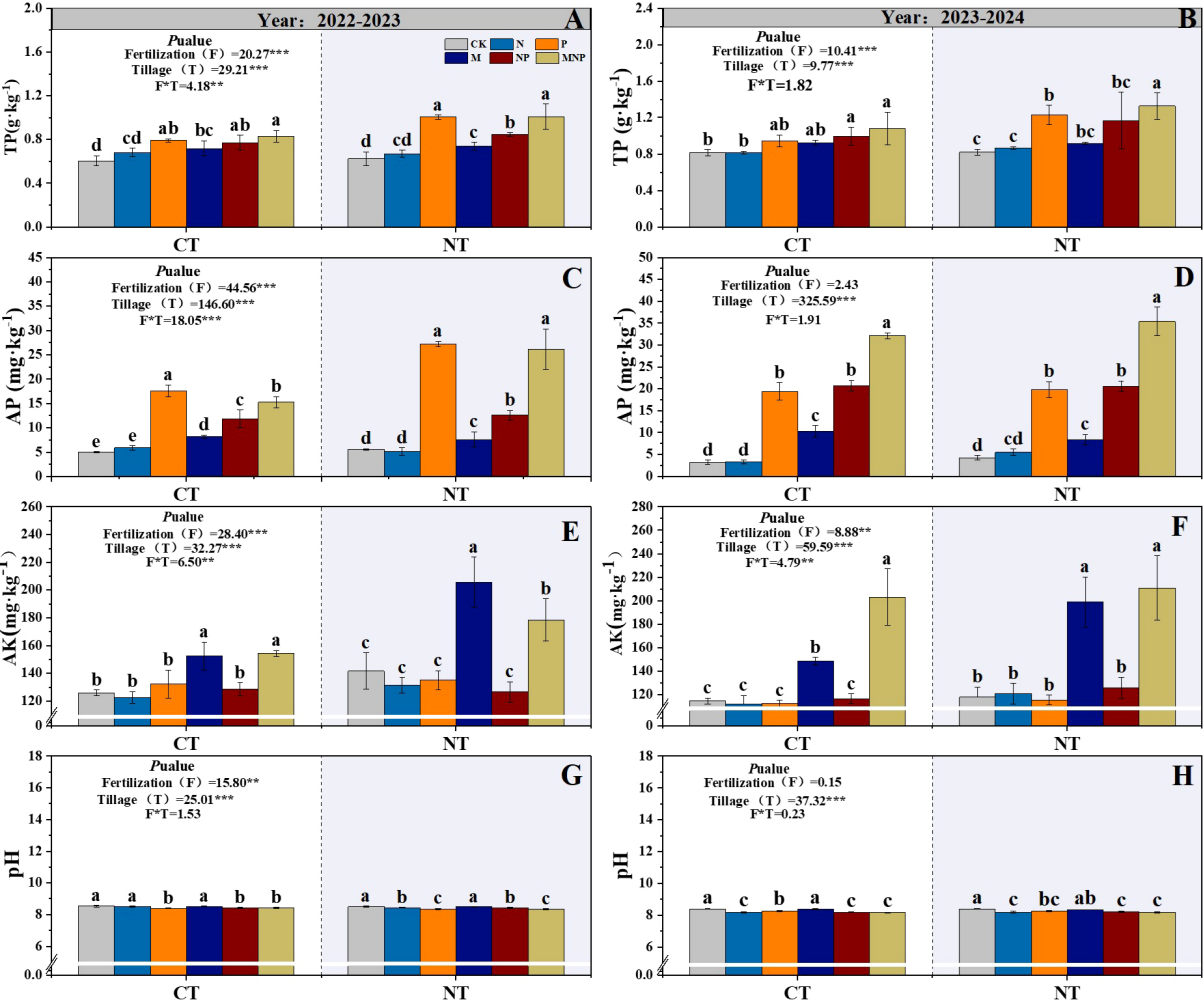
Effects of different treatments on TP **(A, B)**, AP **(C, D)**, AK **(E, F)**, and pH **(G, H)** in Soil during the 2022–2023 and 2023–2024. Significant differences among treatments are indicated in different lowercase letters (P<0.05). **P<0.01, ***P<0.001.

Different fertilization and tillage practices exerted significant effects on soil TP (**Figures 5A, B**), AP (**Figures 5C, D**), and AK (**Figures 5E, F**; *P* < 0.05). Concurrently, tillage practices also markedly altered soil pH (**Figures 5G, H**). NT accumulated TP, AP, and AK, increasing these by 13.25%, 16.75%, and 11.41% respectively compared to CT. Under different fertilization regimes, compared to the NP treatment, the MNP and P treatments resulted in the most pronounced enhancements in soil phosphorus: TP increased by 12.30% and 5.15%, and AP increased by 65.52% and 27.79%, respectively. The MNP and M treatments effectively enhanced soil AK, increasing it by 50.01% and 41.70% respectively relative to the NP treatment. Furthermore, under both NT and CT conditions, all fertilization treatments significantly reduced soil pH compared to the CK.

### 3.4 Bulk density, total soil porosity and field water capacity

**Figure 6:**
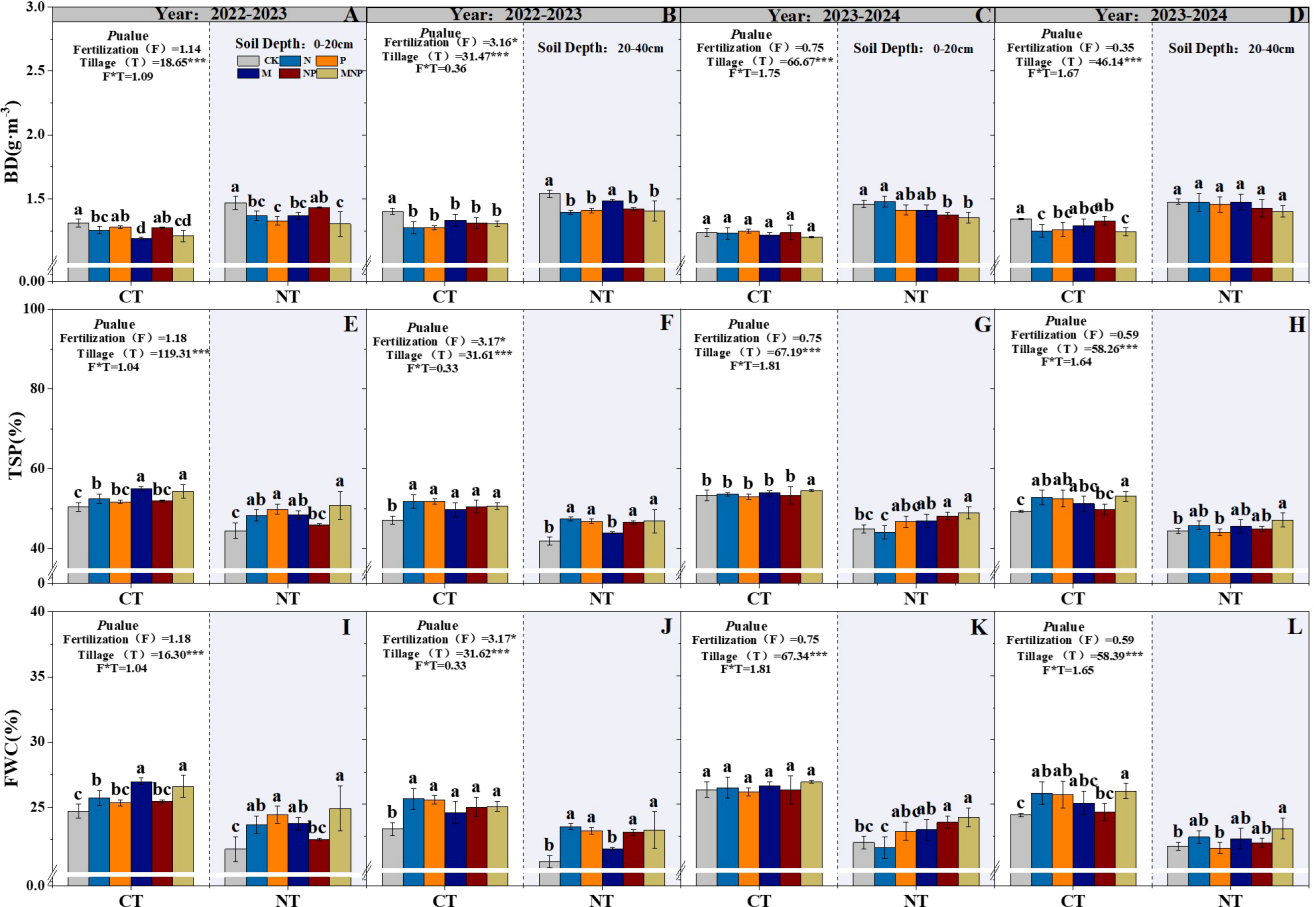
Responses of soil physical properties to different treatments in the 2022–2023 and 2023–2024. **(A–D)** BD, bulk density. **(E–H)** TSP, total soil porosity. **(I–L)** FWC, field capacity. Significant differences among treatments are indicated in different lowercase letters (P<0.05). *P<0.05, ***P<0.001.

Long-term NT significantly increased BD (**Figures 6A–D**) under different fertilization treatments while reducing TSP (**Figures 6E–H**) and FWC (**Figures 6I–L**; *P* < 0.05). The MNP and M treatments effectively lowered BD in the 0–20 cm soil layer by 4.64% and 3.28% respectively compared to the NP treatment. BD increased with soil depth. Concurrently, the MNP and M treatments effectively increased TSP and FWC, though the magnitude of this increase diminished with greater soil depth.

### 3.5 Soil enzyme activities

**Figure 7:**
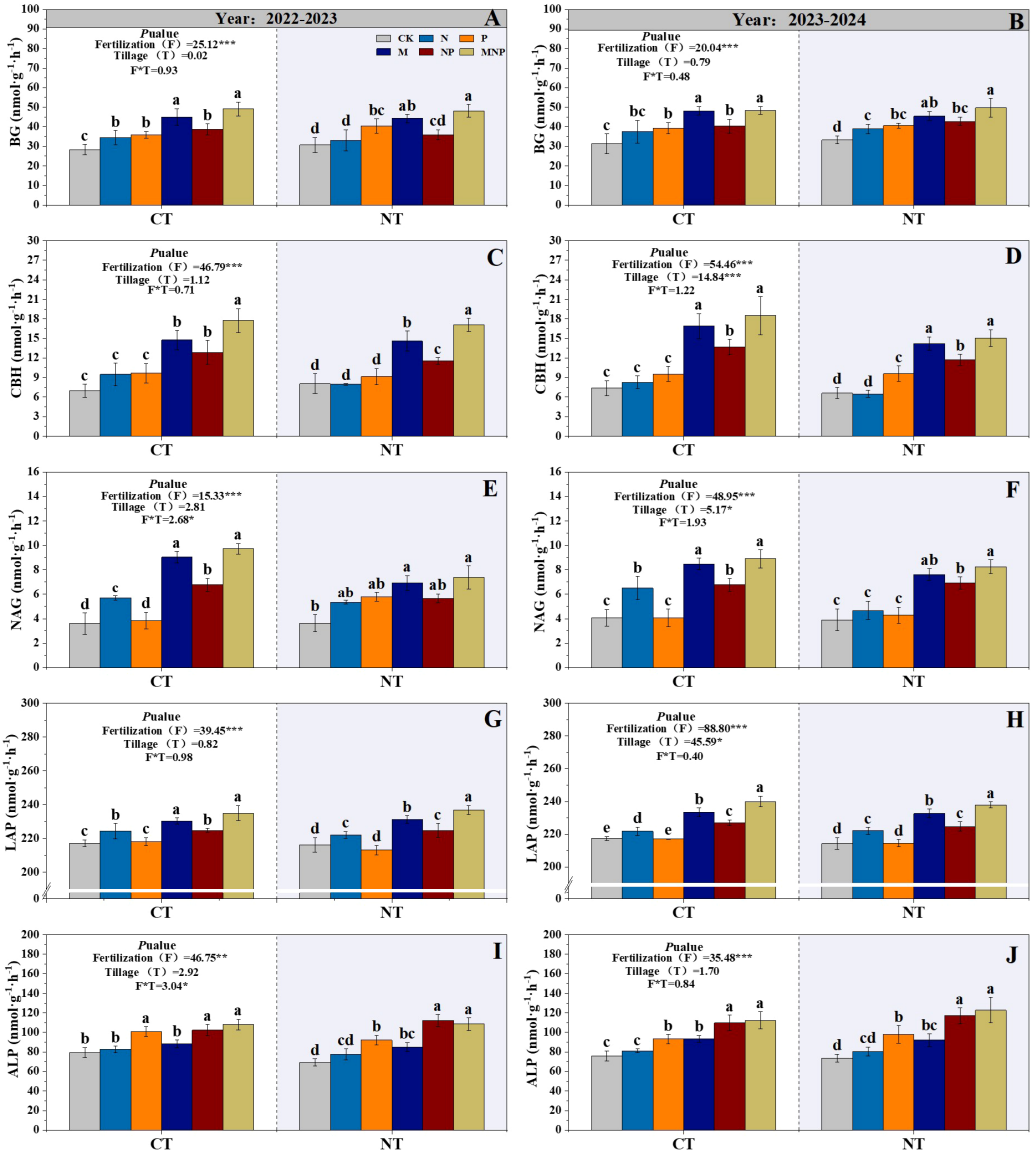
Responses of soil enzyme activity to different treatments in the 2022–2023 and 2023–2024. **(A, B)** BG, β-glucosidase. **(C, D)** CBH, cellobiose hydrolase. **(E, F)** NAG, N-acetylglucosaminidase. **(G, H)** LAP, leucine aminopeptidase. **(I, J)** ALP, alkaline phosphatase. Significant differences among treatments are indicated in different lowercase letters (P<0.05). *P<0.05, **P<0.01, ***P<0.001.

Long-term application of manure alone, as well as combined application of manure and chemical fertilizers, significantly increased soil BG (**Figures 7A, B**), CBH (**Figures 7C, D**), NAG (**Figures 7E, F**) and LAP (**Figures 7G, H**) activities (*P* < 0.05). The impact of different tillage practices on these enzyme activities were relatively minor. Compared to the NP treatment, the MNP and M treatments significantly increased the activities of four soil enzymes: BG increased by 23.94% and 15.96%; CBH increased by 37.28% and 21.35%; NAG increased by 31.04% and 22.68%; and LAP increased by 5.36% and 2.90%, respectively. Specifically, BG and CBH activities exhibited the trend: MNP > M > NP > P > N > CK. Conversely, NAG and LAP activities followed the pattern: MNP > M > NP > N > P > CK. Soil ALP (**Figures 7I, J**) activity was highest under MNP treatment, followed by NP, and significantly higher than other fertilization treatments. However, the ALP activity under P treatment exceeded that under M treatment, increasing by 7.02%. The order of ALP activity was as follows: MNP > NP > P > M > N > CK.

### 3.6 Winter wheat yield and 1000-grain weight

**Figure 8:**
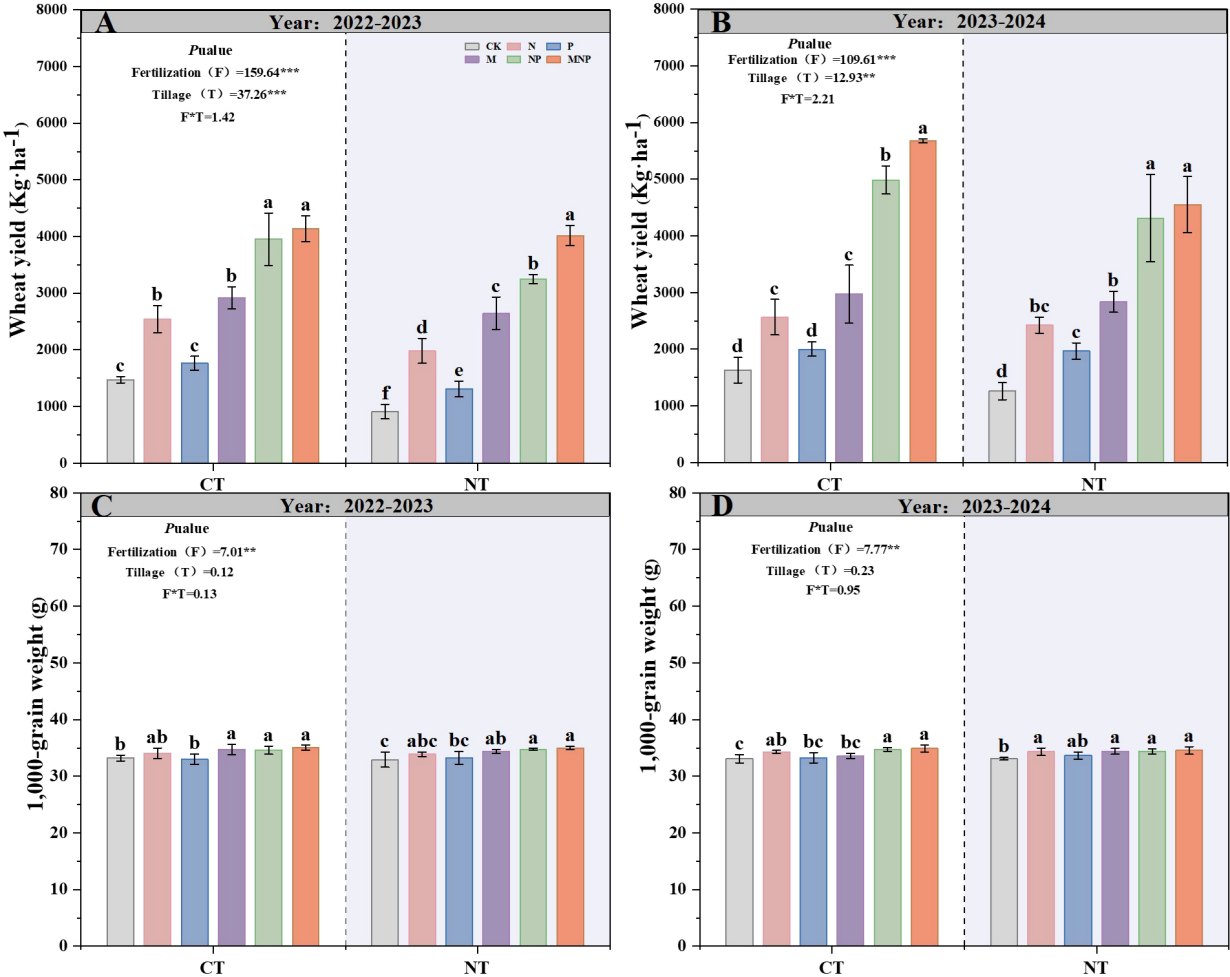
Yield **(A, B)** and 1,000-grain weight **(C, D)** of winter wheat under different treatments in 2022-2023 and 2023-2024. Significant differences among treatments are indicated in different lowercase letters (P<0.05). **P<0.01, ***P<0.001.

The long-term application of manure combined with chemical fertilizers significantly enhances winter wheat yields and 1000-grain weight (**Figure 8**; *P* < 0.05). However, prolonged NT practice result in reduced winter wheat production. Under both tillage regimes, the MNP yielded 9.84% (CT) and 13.40% (NT) higher than the NP. Concurrently, long-term application of manure alone caused yield reductions, decreasing by 34.14% (CT) and 27.45% (NT) compared to the NP treatment. Compared to P, N was more conducive to wheat yield formation. The application of organic fertilizer mitigates the yield constraints associated with long-term NT.

### 3.7 Linkages among soil physicochemical properties, enzyme activities, and winter wheat yield

**Figure 9:**
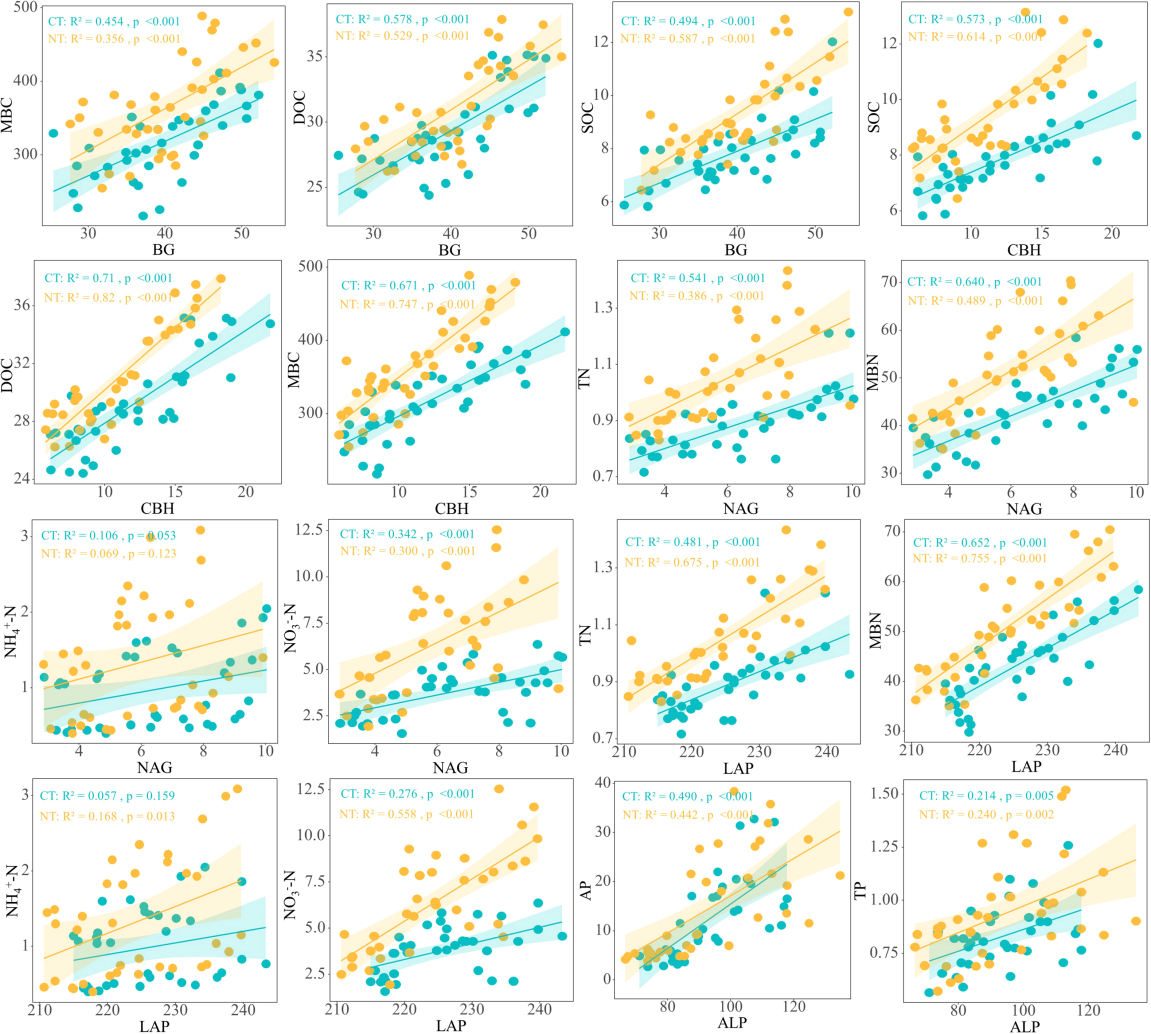
Under two different tillage practices, the soil C, N, and P fractions under each fertilization treatment correspond to the soil enzyme activities.

Analysis of soil C, N, and P fractions and the enzyme activities related to C, N, and P cycling revealed a strong correlation between them; specifically, SOC, DOC, MBC, TN, NO_3_^--^N, and MBN were all significantly stimulated during the decomposition process in NT soil (**Figure 9**; *P* < 0.001).

**Figure 10:**
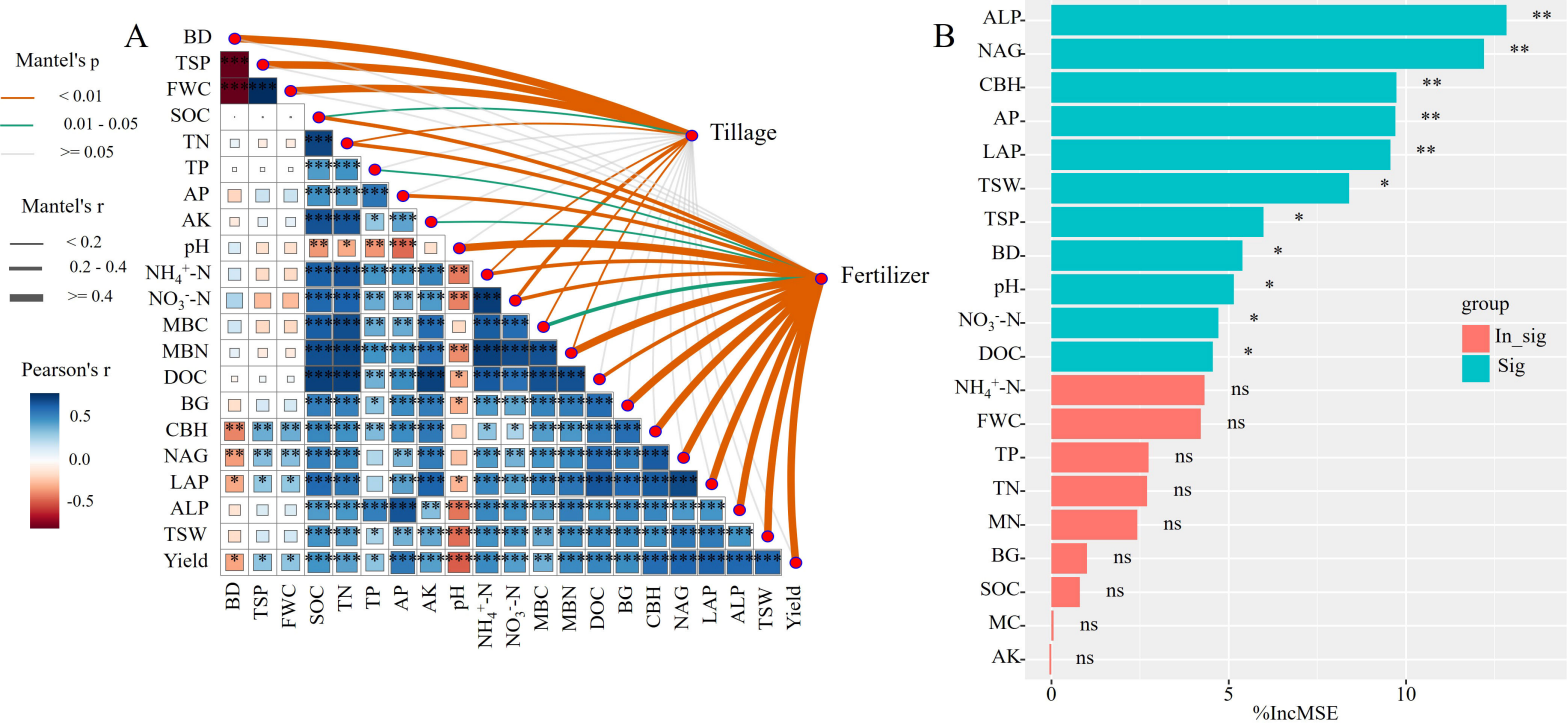
Mantel tests and Pearson correlation matrices revealed the relationships among key soil nutrients, enzyme activities, and grain yield of winter wheat with tillage and fertilizer **(A)**. The lower triangle shows Pearson’s correlation coefficients (r), where color intensity represents the strength and direction of correlations (blue = positive, red = negative). The upper triangle shows Mantel’s test results with connecting lines: orange lines indicate highly significant correlations (*P* < 0.01), blue lines show moderately significant correlations (0.01 < *P* < 0.05), and gray lines indicate no significant correlations (*P* > 0.05). Mantel’s r values are categorized as < 0.2 (thin lines), 0.2–0.4 (medium lines), and > 0.4 (bold lines). Random forests prediction of the relative importance of soil physicochemical properties and enzyme activity on winter wheat yield **(B)**. ***, *P* < 0.001; **, 0.001 < *P* < 0.01; *, 0.01 < *P* < 0.05; and ns *P* > 0.05.

According to the Mantel test results, tillage practices showed significant correlations with multiple soil physicochemical properties and enzyme activities, among which BD, TSP, and FWC exhibited stronger associations (*P* < 0.05). This indicates that long-term NT primarily affects soil physical structure—increasing bulk density while reducing porosity and water-holding capacity—which in turn constrains root penetration and water availability. Fertilization practices were also strongly correlated with a variety of indicators, including pH, MBN, BG, CBH, NAG, LAP, ALP, and TSW. Meanwhile, yield showed a significant positive correlation with all other physicochemical properties except pH and BD (*P* < 0.05). In addition, the correlation between tillage practices and yield was relatively weak, whereas fertilization showed a significant association with yield, indicating that optimizing fertilization measures is more effective in enhancing crop yield. This conclusion highlights the predominance of bottom-up resource-driven regulation over physical constraints in this agroecosystem (**Figure 10A**). To further identify the main drivers, random forest model was applied to predict the key environmental factors influencing winter wheat yield. The model explained 85% of the variation in winter wheat yield. The ranking of environmental factors revealed that ALP was the primary predictor of yield, followed sequentially by NAG, CBH, AP, LAP, TSW, TSP, BD, pH, NO_3_^--^N, and DOC (**Figure 10B**). This is attributable to the strong correlations between ALP, NAG, CBH and soil nutrients (e.g., SOC, TN, MBC, MBN), and consequently, the high importance assigned to these enzymes likely reflects a surrogate effect.

**Figure 11:**
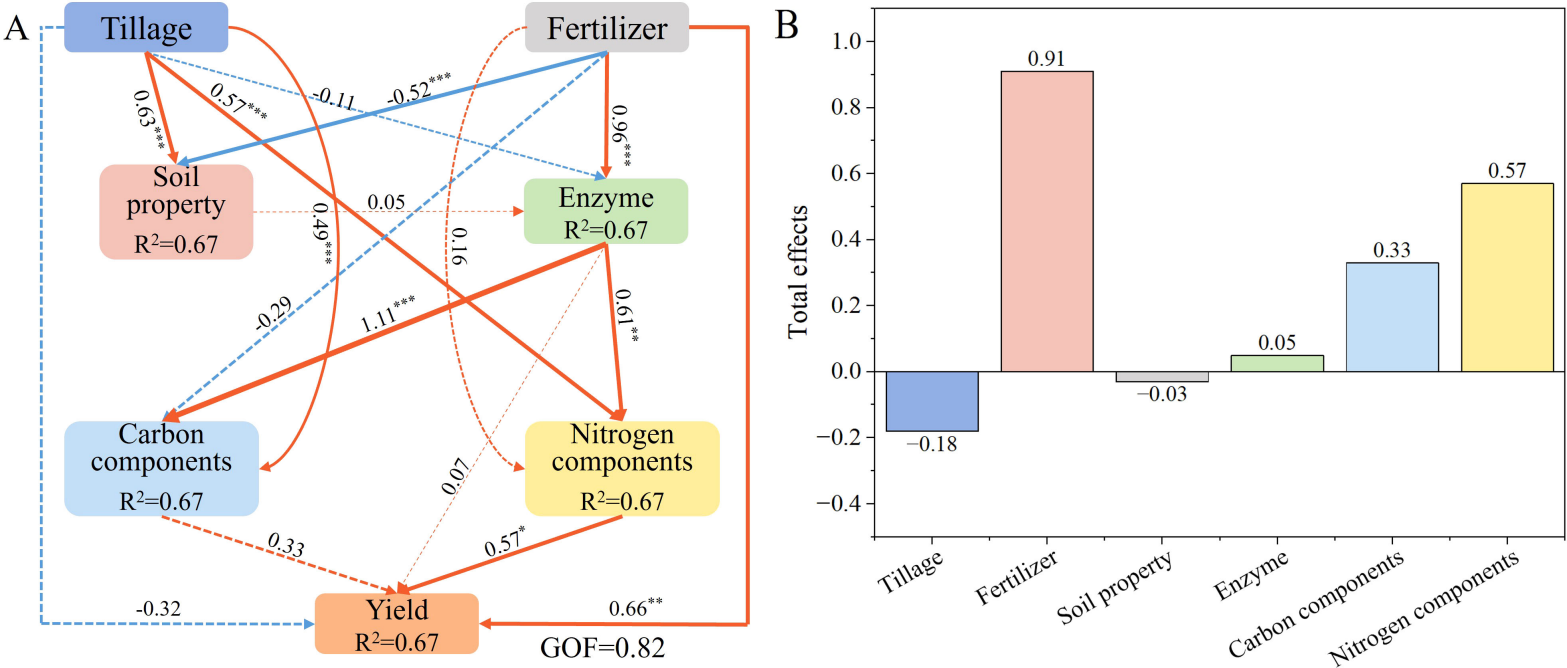
PLS-PM depicts factors influencing yield through direct and indirect pathways **(A)**. Orange arrows represent positive effects, while blue arrows represent negative effects; solid lines indicate significant effects, and dashed lines indicate non-significant effects. Numbers beside the arrows indicate standardized path coefficients (***, *P* < 0.001; **, 0.001 < *P* < 0.01; *, 0.01 < *P* < 0.05). R² indicates the proportion of variance in the dependent variable explained by the model. Four latent variables were included in the model to examine their effects on yield. Soil properties were assessed through soil pH and BD. Enzyme activities were represented by BG, CBH, NAG, LAP, and ALP. Carbon components were characterized by SOC, MBC, and DOC. Nitrogen components were described by TN, MBN, NH_4_^+^-N), and NO_3_^--^N. A bar chart visualizes total effects based on PLS-PM **(B)**.

Further analysis using partial least squares indicated that fertilization was the most important factor affecting winter wheat yield, followed by nitrogen components and carbon components. Overall, manure application was found to regulate soil enzyme activities, thereby influencing carbon and nitrogen components, and ultimately increasing crop yield (**Figure 11**). Manure provides abundant organic substrates that stimulate soil microorganisms to secrete extracellular enzymes (e.g., CBH involved in carbon cycling and NAG involved in nitrogen cycling). These enzymes accelerate the decomposition of organic matter, converting organically bound carbon and nitrogen into plant-available forms (e.g., NH_4_^+^, NO_3_^-^, and labile carbon). This enzyme-mediated activation process enhances the synchrony between soil nutrient supply and crop demand, thereby promoting nutrient uptake and biomass accumulation, and ultimately leading to increased yield.

## 4 Discussion

The soils in the Loess Plateau region of northwest China are typically characterized by silty loam texture and low soil organic matter content, making them highly susceptible to structural degradation and the loss of aggregate stability caused by heavy rainfall and inappropriate tillage practices. This structural deterioration leads to a soil pore system dominated by ineffective, fine capillary pores, significantly weakening water infiltration and retention capacity. Consequently, this exacerbates seasonal drought, severe soil and water erosion, and the continuous decline of soil fertility. These factors constitute the core bottlenecks constraining the sustainable development of rainfed agriculture in this region (Meng et al., 2021; Zhao et al., 2026). Therefore, improving soil structure-particularly by promoting the formation of stable aggregates and enhancing organic matter content-is crucial for strengthening soil erosion resistance, improving water use efficiency, and reversing land degradation.

### 4.1 Effects of NT and organic fertilizer on soil carbon fractions and related enzyme activities

The results of this study indicate that NT and organic fertilizer application significantly affect soil carbon fractions (SOC, DOC, MBC). NT significantly increased SOC, DOC, and MBC. By minimizing soil physical disturbance, NT preserved soil aggregate structure, thereby reducing the mineralization and decomposition rate of organic carbon and promoting SOC accumulation. Simultaneously, it provided a more stable microenvironment for microorganisms, which facilitated the maintenance and increase of MBC (Luo et al., 2016; Sharma et al., 2016). Furthermore, the increase in DOC may be related to the enhanced surface residue cover under NT, the enrichment of root exudates in the topsoil, and the accumulation of fine particles and organic matter in the surface layer (Chan et al., 2002; Zhang et al., 2007). In contrast to NT, the improvement in carbon fractions by organic fertilizer (whether applied alone or in combination) is primarily based on direct nutrient input. Organic fertilizer itself is rich in readily decomposable organic carbon, and its application directly and substantially expands the SOC and DOC pools. Additionally, organic fertilizer contains a large amount of water-soluble organic compounds, which directly contribute to the soil DOC pool and serve as the most rapidly available energy substrates for microbial activity (Ni et al., 2025; Zhao et al., 2018). This confirms the significant increase in DOC under organic fertilizer application observed in this study and directly explains why MBC also increased significantly under organic fertilizer treatments.

In addition, the application of organic fertilizer significantly increased the activities of BG and CBH. These two enzymes are key catalysts for degrading cellulose-based materials-important carbon sources in soil. The direct driver behind this enhancement in activity is the abundance of substrates such as cellulose introduced through organic fertilizer input, which triggers the “substrate-induced effect” that stimulates microbial synthesis of the corresponding enzymes (Moorhead et al., 2016). The increased enzyme activity, in turn, accelerates the decomposition of complex carbon components in organic fertilizer. On one hand, this process releases more DOC. On the other hand, it provides energy for microbial growth, forming a positive feedback loop: substrate increase leads to enhanced enzyme activity, which accelerates carbon transformation, resulting in increased microbial biomass (Dotsenko et al., 2018; Chang et al., 2007). However, NT treatment reduced the activities of BG and CBH. This is because while NT protects the physically stable carbon pool, it also limits the incorporation of fresh plant residues (such as straw) into the soil, thereby reducing the distribution of carbon substrates in the plow layer that could stimulate the activity of specific hydrolytic enzymes (Song et al., 2008; Xuan et al., 2009). Microbial communities may shift toward utilizing other available carbon sources, leading to a decrease in the synthesis of enzymes associated with decomposing fresh residues (e.g., BG, CBH) (Mathew et al., 2012). Therefore, the increase in carbon under NT is primarily attributed to accumulation resulting from physical protection. Furthermore, the changes in soil carbon fractions under NT can be further understood through the lens of microbial carbon use efficiency (CUE). Under NT conditions, reduced physical disturbance preserves aggregate structure, which physically prevents microbial access to organic carbon, thereby decreasing substrate availability for decomposition. This condition may increase apparent CUE, because under substrate-limited conditions, microbes are forced to utilize carbon more efficiently, channeling a greater proportion of assimilated carbon into MBC. This explains our finding that NT significantly increased MBC despite reduced soil enzyme (BG, CBH) activities.

### 4.2 Effects of NT and organic fertilizer on soil nitrogen fractions and related enzyme activities

This study found that both NT and the application of organic fertilizer (compared to conventional chemical fertilizer) increased soil TN, NO_3_^--^N, NH_4_^+^-N, and MBN. This indicates that both practices share a positive effect in enhancing the soil total nitrogen pool, improving the supply of available nitrogen, and promoting biological nitrogen immobilization. Although NT increased total nitrogen fractions, it may simultaneously increase the risk of nitrogen loss via denitrification and nitrate leaching. Under NT, the lack of soil mixing and the formation of continuous macropores (e.g., earthworm burrows, root channels) can create preferential flow paths, potentially accelerating nitrate leaching during heavy rainfall events. The application of organic fertilizer alone led to a decrease in NH_4_^+^-N and NO_3_^--^N. In contrast, the application of nitrogen fertilizer alone maintained higher levels of NO_3_^--^N, NH_4_^+^-N, and MBN. Concurrently, organic fertilizer application significantly increased the activity of key nitrogen-cycling enzymes (NAG and LAP) and lowered soil pH, whereas NT suppressed the activity of these two enzymes. Contrary to some studies that reported increased enzyme activities under NT, this discrepancy may be attributed to differences in residue management—our study involved complete aboveground straw removal, whereas many positive reports on NT enzyme activity often include straw retention. Without straw input, NT may limit substrate availability for enzyme production, leading to suppressed activity. Sole application of organic fertilizer typically has a higher carbon-to-nitrogen ratio. When organic fertilizer is incorporated into the soil, the high content of readily decomposable carbon provides ample energy for microorganisms, but the corresponding nitrogen is relatively insufficient (Mustafa et al., 2020). To meet their growth demands under these conditions, microorganisms strongly immobilize the existing inorganic nitrogen (NH_4_^+^-N and NO_3_^--^N) in the soil, converting it into MBN, which leads to a short-term decline in NH_4_^+^-N and NO_3_^--^N (D’Amours et al., 2023; Ma et al., 2024). Conversely, the sole application of nitrogen fertilizer or the combined application of organic and chemical fertilizers directly supplies sufficient available nitrogen, alleviating microbial nitrogen limitation and thus maintaining a higher pool of available nitrogen. Furthermore, the addition of organic fertilizer serves as a direct substrate, inducing microorganisms to secrete NAG and LAP to decompose these complex organic nitrogen compounds (Cotrufo et al., 2015; Giannetta et al., 2018; Yu et al., 2026). Therefore, the high enzyme activity under organic fertilizer treatments indicates robust nitrogen mineralization potential. However, this does not necessarily translate into immediate inorganic nitrogen release. Under conditions of a high carbon-to-nitrogen ratio, the nitrogen released via mineralization may be rapidly immobilized by microorganisms in close proximity, resulting in an increase in MBN (Marx et al., 2002; Sharma et al., 2016). Simultaneously, the application of organic fertilizer increases the production of organic acids from organic matter decomposition and hydrogen ions generated during nitrification processes, thereby lowering soil pH. The acidified environment may inhibit the activity of nitrifying bacteria, consequently affecting the rate of NO_3_^--^N production (Schroder et al., 2011).

### 4.3 Coordinated optimization of carbon and nitrogen management via NT and combined organic-inorganic fertilization

On the one hand, no-tillage reduces soil disturbance → preserves aggregate structure → limits substrate accessibility → reduces hydrolytic enzyme (BG, CBH, NAG) activities → decreases decomposition rates → increases carbon and nitrogen through physical accumulation → reduces yield. On the other hand, manure input provides readily decomposable organic substrates → stimulates microbial growth (increasing MBC and MBN) → induces extracellular enzyme (CBH, NAG, ALP, LAP) secretion → accelerates decomposition and nutrient mineralization → increases available nutrients (DOC, AN, NN, AP) → enhances yield. Therefore, combining the physical protection advantages of NT with the biochemical priming effects of organic fertilizer may represent the optimal strategy for achieving a balance between carbon sequestration and productivity. In terms of nitrogen management, integrating the nitrogen conservation benefits of NT with the nitrogen transformation advantages of combined organic and inorganic fertilization is key to simultaneously improving nitrogen use efficiency, ensuring crop demand, and enhancing soil fertility. This requires the precise application of chemical nitrogen fertilizers based on the carbon-to-nitrogen ratio characteristics of organic amendments in agricultural production, thereby regulating the microbial “immobilization-mineralization” balance of nitrogen and optimizing the timing of nutrient supply. Future research could further focus on the long-term co-evolution of soil carbon and nitrogen fraction structures and microbial functional communities under this integrated management model.

### 4.4 Study limitations

A limitation of this study is that the tillage treatments differed not only in soil disturbance regime but also in straw (stubble) placement: stubble was incorporated into the soil under CT whereas it was left on the soil surface under NT. Consequently, the observed differences in soil properties, enzyme activities, and yield between CT and NT reflect the combined effects of tillage and straw management, rather than the sole effect of tillage practice. Future studies with additional factorial treatments (e.g., CT with straw removal or surface retention, NT with straw incorporation) are required to disentangle the individual contributions of tillage and straw placement.

## 5 Conclusion

In summary, the combination of NT and organic fertilizer improved soil physical structure and regulated the soil acid-base environment. These improvements in fundamental soil conditions, coupled with the direct substrate effect of organic fertilizer input, collectively stimulated key soil enzyme activities. The enhancement of enzyme activities, in turn, drove the transformation and retention of soil carbon and nitrogen fractions, significantly increasing the contents of SOC, DOC, TN, and MBN. Ultimately, the systematic improvement in soil fertility promoted crop yield formation. Among these factors, the continuous input of organic fertilizer and the increased availability of nitrogen fractions were the most critical contributors to the yield gain. In long-term NT farmland, the combined application of organic fertilizer and inorganic nitrogen can effectively offset the yield reduction caused by NT while maintaining the soil conservation benefits of NT, providing a scientific basis for promoting conservation agriculture and integrated organic-inorganic fertilization in arid regions. This study has two limitations. First, the lack of stratified sampling precluded the analysis of vertical stratification characteristics in the NT system. Second, differences in residue placement (i.e., straw incorporation into the soil under conventional tillage versus straw retention on the surface under NT) may have introduced confounding effects. Future research should employ stratified sampling and factorial designs to disentangle the independent contributions of tillage, residue management, and organic fertilization.

## Data Availability

The raw data supporting the conclusions of this article will be made available by the authors, without undue reservation.
